# Selected Factors of Quality of Raw Salami‐Type Sausage Made From Meat of Two Rare Native Cattle Breeds: Polish Red and White‐Backed

**DOI:** 10.1002/fsn3.71733

**Published:** 2026-04-15

**Authors:** Ewelina Węsierska, Małgorzata Pasternak, Katarzyna Niemczyńska‐Wróbel, Jacek Słupski

**Affiliations:** ^1^ Department of Infectious Diseases and Public Health University of Agriculture in Krakow Krakow Poland; ^2^ Department of Plant Products Technology and Nutrition Hygiene University of Agriculture in Krakow Krakow Poland

**Keywords:** native cattle, Polish Red, quality, salami, White‐Backed

## Abstract

The aim of the study was to evaluate selected quality‐related factors of salami‐type sausages as nutritional and chemical composition, texture attributes, safety and microbiological status of dry‐cured, salami‐type sausage spontaneously fermented, produced from the meat of Polish Red (PR; *polska czerwona*) and White‐Backed (WB; *białogrzbieta*) native cattle breeds. The work involved the evaluation of the following properties: chemical (basic chemical composition, biogenic amine content), physicochemical (pH, water activity a_w_, color and texture parameters) and microbiological (lactic acid bacteria LAB and coagulase‐negative staphylococci CNS). Ripening time, pH and a_w_ affected the populations of LAB and CNS and determined the redness and brightness of the final products. Sausages produced from PR and WB cattle meat did not differ in their basic chemical composition and therefore exhibited comparable energy values. Significant differences were observed between PR and WB sausages in color parameters, particularly redness and lightness as well as in the proportion of lactic acid bacteria. Despite comparable processing conditions, WB sausages showed a significantly higher histamine content, indicating differences in the metabolic activity of the bacteria. The use of black garlic significantly affected the color characteristics of the sausages, resulting in lower lightness, reduced color saturation, and greater overall color deviation. Sausages containing black garlic were characterized by higher hardness and shear force, but lower springiness, despite comparable water, protein, and fat contents. Increased salinity, reduced water activity, and low pH, regardless of garlic type, did not inhibit the growth of LAB but limited the development of CNS.

## Graphical abstract

The meat of Polish Red and White‐Backed is a technologically attractive to produce dry‐cured salami‐type sausages The chemical composition of the meat of Polish red and White‐Backed breeds is safe, although it provides a favorable environment for the development of acidic and denitrifying bacteria. Decarboxylation of histidine and tyrosine indicates a high metabolic activity of microbiota under comparable conditions for all sausage types, that is, water content, water activity and pH.

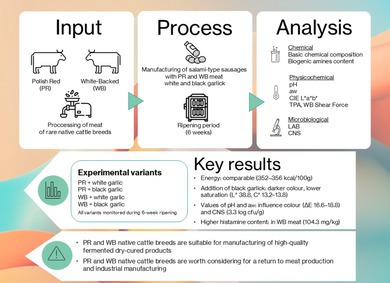

## 1 Introduction

A current trend is the search for foods with health‐promoting properties, such as antioxidant activity, blood pressure reduction, improved nutrient absorption, and mitigation of changes caused by fatigue, stress, smoking, and adverse environmental factors (Decker and Park 2010; Jiménez‐Colmenero et al. 2010; Fernandes et al. 2017). Dry‐cured sausages are a rich source of protein, peptides, exogenous amino acids, and essential fatty acids released during several weeks of fermentation (Arihara 2006; Jiménez‐Colmenero et al. 2010; Marušić et al. 2013; Degnes et al. 2017). Furthermore, fermented sausages provide an environment for the development of halotolerant strains of lactic acid bacteria (LAB), including probiotic strains, as well as coagulase‐negative staphylococci (CNS), which stimulate the immune system (Baka et al. 2011; Danilović et al. 2011; Ravyts et al. 2012; Rossi et al. 2023). The activity of technologically desirable microbiota and the catabolism of amino acids and fatty acids result in the formation of flavor precursors and indirectly affect physical quality characteristics (color and texture) (Brewer and Novakofski 2008; Martin et al. 2007; Romero et al. 2013; Mati et al. 2015; Mora et al. 2015; Stadnik and Kęska 2015; Martínez‐Arellano et al. 2016; Degnes et al. 2017; Zhou et al. 2017; Węsierska, Sobolewska‐Zielińska, et al. 2021). Therefore, to meet the expectations of consumers looking for meat products that are nutritionally attractive, convenient to use, and produced using technology like traditional methods, it is worthwhile to draw on the technology of dry‐cured sausages made from high‐quality meat of native animal breeds and enriched with spices valued for their health‐promoting effects. The addition of black garlic enhances the product with a sweet flavor accented by a slight hint of liquorice root and increases its attractiveness in terms of health‐promoting properties. The well‐known health‐promoting properties of black garlic are associated with its high content of natural antioxidants (Bae et al. 2014; Jeong et al. 2016; Kang 2016; Kimura et al. 2017). These antioxidant properties help maintain high fat quality throughout the ripening period and are valuable in preventive health care (Wójciak and Dolatowski 2012; Rossi et al. 2023). However, dry‐cured products, due to their higher sodium content resulting from the salting or curing process and the accumulation of biogenic amines (BAs) arising from the activity of acidifying microbiota, may be perceived as unsuitable for individuals with chronic non‐communicable diseases. The xenobiotic transformation of microbiota depends on the biodiversity and abundance of indigenous bacterial populations (Ordóñez et al. 1999; Lebert et al. 2007; Leroy et al. 2010; Zhou et al. 2017). Excessive growth of bacteria capable of decarboxylating free amino acids can lead to increased levels of BAs such as putrescine, cadaverine, tyramine, phenylethylamine, and histamine (Latorre‐Moratalla et al. 2008; Maintz et al. 2008; Olivares et al. 2009; Baka et al. 2011; Danilović et al. 2011; Ravyts et al. 2012; Alvarez and Moreno‐Arribas 2014; Berardo et al. 2017; Węsierska, Sobolewska‐Zielińska, et al. 2021). In fact, the high proteolytic activity of endo‐ and exogenous enzymes, the optimal protein‐to‐fat ratio, and the favorable water content and activity promote decarboxylase action and the production of BAs (Romero et al. 2013; Berardo et al. 2017; Zhou et al. 2017; Rossi et al. 2023). The group of LAB with well‐developed amino acid decarboxylation abilities includes even strains used as starter cultures (*Levitlactobacillus brevis*, *Lentilactobacillus buchneri, Latilactobacillus curvatus*). Bacteria such as *Bacillu*s sp., *Citrobacter* sp., *Clostridium* sp., *Klebsiella* sp., *Proteus* sp., *Pseudomonas* sp. and *Lactococcus* sp. are also capable of producing BAs. Studies have shown that the production of BAs is not a widespread property among staphylococci (Bartkiene et al. 2017). Only 3.6% of coagulase‐negative *Staphylococcus* strains isolated from cured meats are able to produce certain BAs, including histamine, putrescine, cadaverine, and tyramine (Landeta et al. 2007; Hospital et al. 2015). Coagulase‐negative staphylococci prevalent in spontaneously fermented sausages include *Staphylococcus equorum* , *S. xylosus* , *S. saprophyticus* and *S. warneri* (Hospital et al. 2015; Węsierska 2019). Therefore, it is essential to study technologies that incorporate “new‐old” raw materials, considering not only their nutritional value and technological quality but also their safety with respect to the presence of BAs. The aim of this study was to compare the quality and safety of dry‐cured, salami‐type sausages made from the meat of two native cattle breeds: Polish Red and White‐backed. The formation of technological, nutritional, and microbiological quality and safety during the 6‐week ripening was analyzed with particular attention to biogenic amine content.

## 2 Materials and Methods

### 2.1 Manufacturing

The spontaneously fermented sausages were produced using meat from Polish Red (PR) and White‐Backed (WB) native cattle breeds, with the addition of white (w) and fermented black (b) garlic (Table 1). Four variants of salami‐type sausages were produced: PR × w, PR × b, WB × w, WB × b. All variants were monitored throughout the entire ripening period. The production process was carried out in one of the meat‐processing plants in the Lesser Poland Voivodeship (*Małopolska*), in ripening chambers, following established procedures for the selection of meat and fat raw materials, spices, and additives, as well as technology appropriate for this type of product.

**TABLE 1:** Technology of dry fermented sausages (the same proportion of raw materials for each variant per 100 kg of stuffing).

Basic raw material (per 100 kg)	Quantities	Grinding
Meat and fat raw materials		
Polish Red or White‐Backed cattle breeds beef class I	70 kg	20 ± 5 mm
Pork bacon without bones	30 kg	20 ± 5 mm
Spices, additives (per 100 kg of basic raw material)		
Curing salt	2.4 kg	
Ground black pepper, sugar, sweet red pepper	Each 0.3 kg	
White or black fermented garlic	0.3 kg	
Auxiliary materials (per 100 kg of basic raw material)		
Vapor‐permeable casing fibrous 55 mm	70 m	
Summary	103.6 kg	Pre‐ripening mass
	73 kg	Mass of product
	1.54%	Machine losses
	28.43%	Losses due to drying

Once the ingredients had been received and their quality verified, the next step was to combine them during the processing stage. The quantitative composition of raw materials and additives in each batch was the same for all four variants. Chilled meat pieces (below −4°C) and frozen fat tissues/bacon (below −8°C) were chopped and ground using a grinder. The meat and bacon were minced into Ø 20 mm pieces and cured for 48 h at 4°C−6°C with 24 g/kg of curing salt (min. 98.4% NaCl, 0.6% NaNO_2_; Anna, Poland). After curing was completed, the meat and bacon were minced once more, this time into pieces Ø 5 mm, mixed with spices (sugar 3 g/kg, pepper 3 g/kg, sweet red pepper 3 g/kg, white or black fermented garlic 3 g/kg), and filled into casings (Handtmann VF 50, Germany; Fabios, Poland). The batter was stuffed at 0°C–1°C into casings. The proportion of beef meat to pork was 70:30. The main objectives of fermentation were to stimulate microbial activity and to achieve an appropriate pH decrease. The sausages were hung in air‐conditioned ripening chambers equipped with computerized control of temperature, relative humidity (RH), and air velocity. During the first 2 days (primary fermentation), the sausages ripened at 18°C and 90% RH. Subsequently, during the drying stage, the temperature and humidity in the chamber were reduced to 16°C and 75% RH. During ripening, sausages should: (1) exhibit a pH decrease resulting from the production and accumulation of lactic acid, (2) develop an attractive color due to the accumulation of nitrosylmyoglobin, (3) acquire the expected texture as a result of the combined effects of temperature, RH, and pH, and (4) develop a characteristic aroma due to endo‐ and exogenous enzymes. The sausages prepared for retail sale were subjected to the same quality control procedures as all other products of the plant, in accordance with food safety requirements. Four separate batches (PRw, PRb, WBw, WBb) were used in three production runs. From each batch, three 1‐kg sausage samples were collected and analyzed as replications (3 production runs × 3 one‐kilogram replications per batch). The above‐mentioned samples were prepared immediately after collection for chemical, physicochemical, and microbiological analysis. Samples were collected during ripening (after 0, 2, and 4 weeks) and immediately after its completion (after 6 weeks).

### 2.2 Analysis

#### 2.2.1 Basic Chemical Composition and Energy Value

The moisture and ash content were determined by drying samples to a constant weight (ISO 1442:2000, ISO 936:2000). The protein content was determined using the Kjeldahl method (KjelMaster K‐375, Büchi, Switzerland) with a conversion factor of 6.25 (PN‐75‐A‐04018:1975/Az3:2002). Fat content was evaluated using the Soxhlet method (SOXTEC HTZ‐2, Tecator, Sweden) with ethyl ether extraction (ISO 1444:2000), and the salt content was determined using Mohr's method (PN ISO 1841‐1:2002). The energy value of meats was calculated using Atwater specific factors (FAO 2003).

#### 2.2.2 Color Characteristics

The color was assessed according to the CIEL*a*b* standard (Minolta CR200b analyzer calibrated according to the white reference standard: L* = 94.2; a* = 0.3133; b* = 0.320; Wyszecki and Stiles 1982). The following color descriptions were assessed: saturation C* = (a*^2^ + b*^2^)^1/2^, hue *H* = tan‐1 b*/*a*, and delta ∆E =$\;\sqrt{{\left(∆L^{*}\right)}^{2}+{\left(∆a^{*}\right)}^{2}+{\left(∆b^{*}\right)}^{2}}$. The ∆E values were to indicate color deviation that was not visible, with a difference in the measurement accuracy limit (0.00–1.00), slight deviation, recognizable by an expert (1.01–2.00), medium, recognizable by an inexperienced observer (2.01–3.50), significant (3.51–5.00), large (5.01–7.50) and very evident (> 7.5).

#### 2.2.3 Texture Characteristics

The Texture Profile Analysis (TPA) was conducted as described by Breene and Barker (1975) using a TA‐XT2 texture analyzer (Stable Micro Systems, UK) equipped with a cylindrical probe (14 mm diameter, 15 mm length). Samples were compressed twice, parallel to the fiber direction, to 70% of their original size. The Warner‐Bratzler Shear Force (WB shear force) was determined using seven cylindrical samples (14 mm diameter and 15 mm length). Measurements were carried out with a TA‐XT2 texture analyzer (Stable Micro Systems, UK) fitted with a WB shear blade with a triangular cut‐out. Results were processed using Stable Micro Systems Texture Expert software for Windows, version 1.05 (Stable Micro Systems, UK). The pH was measured with a CP‐411 pH meter and PP‐3 electrode (Elmetron, Poland) in water homogenate (meat: water 1:3). Water activity (aw) was determined using the LabMaster‐aw meter (Novasina, Switzerland), following the manufacturer's instructions.

#### 2.2.4 Quantitative Composition of Acidifying and Denitrifying Bacteria

Lactic acid bacteria (LAB)—cultured on MRS Agar (Biomérieux), with pH adjusted to 5.4 using acetic acid; incubation at 30°C for 24–48 h in an anaerobic chamber with a CO_2_‐enriched atmosphere (20%; Sheldon Manufacturing Inc.) (ISO 15214:2002). Coagulase‐negative staphylococci (CNS)—cultured on Baird Parker Agar Base supplemented with yolk emulsion and sodium tellurite (Biomérieux); isolates were classified as coagulase‐negative based on coagulase activity; incubation at 37°C for 24 h (ISO 6888‐1:2022).

#### 2.2.5 Biogenic Amines Content

Biogenic amines (BGAs) were analyzed using HPLC (Innocente et al. 2007), with minor modifications. A 10‐g meat sample was mixed with 15 mL of 6% trichloroacetic acid (TCA), homogenized for 2 min (1000 rpm, Vortex) and centrifuged at 14,000 × g for 20 min at 4°C (MPW Med. Instruments, Poland). The resulting supernatant was collected and filtered through Whatman filter paper No. 1. The residue was reextracted with the addition of 15 mL of fresh 6% TCA. The combined supernatants were then brought to a final volume of 50 mL with 6% TCA. The derivatisation process was carried out by mixing 1 mL of the extract with 1 mL of a dansyl chloride solution in acetone (10 mg/mL) and 0.5 mL of saturated NaHCO_3_ solution. The mixture was incubated at 40°C for 60 min with occasional shaking in a thermoblock (Dry Block Heater, IKA, Germany). BGAs were extracted twice using 1 mL of diethyl ether for 10 min (22°C). The combined extracts were evaporated to dryness under a nitrogen stream, and the residue was dissolved in 1 mL of acetonitrile. The solution of BGAs standards was prepared by mixing 1 mL of each free base standard solution, containing 0.1 mg mL^−1^ of each BGA, with 5 mL of the dansyl chloride acetone solution. The derivatisation and extraction procedures were performed in the same manner as the samples. Chromatographic separation was performed using the Dionex UltiMate 3000 HPLC apparatus (Thermo Scientific, USA). The separation was carried in a Nova‐Pak reverse phase C18 column, 4 μm particle size, 150 × 3.9 mm (Waters, USA), thermostated at 30°C. The two solvent reservoirs contained the following eluents: (A) acetonitrile, (B) HPLC grade water at a flow rate of 0.8 mL min^−1^. The elution programme was: 65% A and 35% B for 1 min, increasing to 80% A and 20% B for 9 min, increasing to 90% A and 10% for 2 min, increasing to 95% A and 5% B for 4 min, maintaining for 7 min, then returning to 65% A and 35% B, and holding for 5 min. Fluorometric detection was carried out using excitation and emission wavelengths of 340 nm and 530 nm, respectively. The standard solution of eight BGAs was prepared in 0.1 M HCl in a concentration of 1 mg mL for every amine. Each of the following reference substances was dissolved in 10 mL of 0.1 M HCl: tyramine hydrochloride (Sigma‐Aldrich, Switzerland) 12.7 mg, tryptamine hydrochloride (Sigma‐Aldrich, USA) 12.3 mg, histamine dihydrochloride (Sigma‐Aldrich, China) 16.6 mg, putrescine dihydrochloride (Sigma‐Aldrich, Switzerland) 18.3 mg, cadaverine dihydrochloride (Sigma‐Aldrich, Switzerland) 17.1 mg, spermine tetrahydrochloride (Sigma‐Aldrich, Switzerland) 17.2 mg, spermidine trihydrochloride (Sigma‐Aldrich, Switzerland) 17.5 mg and 2‐phenylethylamine hydrochloride (Sigma‐Aldrich, Japan) 13.0 mg. The prepared solution of standards was then diluted 100‐fold. All used reagents were HPLC grade (Sigma‐Aldrich, USA).

#### 2.2.6 Statistical Analysis

The statistical analysis was performed using the Statistica software for Windows, version 13.3 (TIBCO Inc., USA). The effect of the ripening time on the chemical properties was tested using three‐factors analysis of variance: breed (B): PR, WB; ripening time (T): 0, 2, 4, 6 weeks; type of garlic (G): w, b (ANOVA with fixed and orthogonal factors). The statistical tables describe the interactions: B × T, B × G, G × T and B × T × G. The Duncan post hoc tests were used to compare the means at *p < 0.05* as well as the Pearson's correlation coefficient (*r*) was used to test the statistical relationship between two continuous variables.

## 3 Results and Discussion

Traditional salami is a raw, dried product made from beef and pork, with the addition of pork fat, often covered with noble mold strains. This technology is used today to produce Italian Sorrento, Nola, Napolitano, Genoa, Finocchione, Felino, and Hungarian salami. The names of these sausages are associated with the region of production and are often accompanied by PDO (Protected Designation of Origin) or PGI (Protected Geographical Indication) labels. Following the traditional technology, spontaneous fermentation of PC and WB salami sausages was carried out and compared in terms of quality characteristics. Differences between cattle breeds (PR vs. WB) and garlic type (white vs. black) were observed for selected quality parameters and are described below using ANOVA.

### 3.1 Basic Chemical Composition and Energy Value of Salami‐Type Sausages Made From Meat of Two Rare Cattle Breeds

Table 2 presents the results obtained for salami‐type sausages produced from Polish Red (PR) and White‐Backed (WB) cattle meat with white (w) and black (b) garlic. ANOVA showed that dry‐cured salami‐type sausages made from PR and WB meat did not differ in water, protein, fat, ash, and salt content; thus, their nutritional value was comparable (352 and 356 kcal/100 g). During the 6‐week ripening, the chemical composition of the sausages changed significantly (*p* < 0.05), due to the successive drying and the reduction of water content. Protein, fat, ash, and salt content increased, reaching standard levels in ready‐to‐eat products. An interaction analysis showed the influence of breed, garlic, but particularly ripening time on ash content (*p* < 0.01). Increasing fat content correlated with decreasing water content throughout ripening, that is, at the start of production (*r* = −0.93), in 2nd (*r* = −0.70), 4th (*r* = −0.60) and the 6th week (*r* = −0.76), as well as the salt with water content in the 6th week (*r* = −0.69).

**TABLE 2:** Influence of breed, ripening time (week) and type of garlic on basic chemical composition (%) and energy value (kcal/100 g) of dry fermented sausages made from Polish Red and White‐Backed cattle meat with white and black garlic (ANOVA).

	Breed (B)		Ripening time (T)				Garlic (G)		Interactions			
	PR	WB	0	2	4	6	w	b	B × T	B × G	G × T	B × T × G
Moisture	50.70	51.10	59.20^a^	52.69^b^	50.08^c^	41.61^d^	50.86	50.93				
Protein	19.98	20.32	17.42^a^	19.31^b^	20.12^c^	23.76^d^	20.02	20.28				
Fat	23.96	23.34	19.62^a^	23.25^b^	24.89^c^	26.85^d^	23.69	23.62				
Ash	2.90	2.98	2.14^a^	2.59^b^	3.09^c^	3.94^d^	2.92	2.96	**		**	**
Salt	2.08	2.01	1.44^a^	1.80^b^	1.98^b^	2.96^c^	2.09	2.00				
Energy	PR: 352 kcal/100 g			WB: 356 kcal/100 g								

*Note:* ^a,b^different letters in the rows indicate significant differences between the means at *p* < 0.05; *significant interaction at *p* < 0.05; **significant interaction at *p* < 0.01.

Abbreviations: PR, Polish Red cattle breed; w/b, white/black garlic; WB, White‐Backed cattle breed.

The varying proportion of water, protein, fat, and salt during ripening shapes the quality of fermented sausages as it affects juiciness, texture, color, and taste (Olivares et al. 2009; Marušić et al. 2013; Degnes et al. 2017; Węsierska et al. 2026). The presence of sodium chloride, due to the technology and safety of fermented sausage production, is so far irreplaceable, as it inhibits the growth of undesirable microorganisms (by decreasing water activity) and improves the organoleptic experience (by flavor enhancement and texture formation) (Ordóñez et al. 1999; Lebert et al. 2007; Leroy et al. 2010; Zhou et al. 2017). Due to their chemical composition, fermented sausages are perceived as foods that supplement the diet with valuable components used by the human body, for example, in tissue reconstruction (Marušić et al. 2013; Dyall 2015; Jiménez‐Colmenero et al. 2015; Heras‐Sandoval et al. 2016), and as a carrier of vitamins A, D, E and K (Goncalves and Amiot 2017; Kindleysides et al. 2017).

### 3.2 Color of Salami‐Type Sausages Made From Meat of Two Rare Cattle Breeds

The color of the sausages was shaped by factors related to genetic variation (breed), ripening time and the addition of two types of garlic (Table 3). Salami‐type sausages from the meat of PR and WB cattle breed differed in redness (a*) and brightness (luminance) (L*) (*p* < 0.05). The color changed during ripening (*p* < 0.05) due to drying. The sausage stuffing converted into darker color. Furthermore, the proportion of redness decreased (*p* < 0.05). Sausages made with the addition of black garlic were characterized by darker color (*p* < 0.05) and lower saturation (Table 4). Moreover, ΔE in this group was greater. Evaluating the quality of the salami‐type sausages produced from the meat of PR and WB cattle breeds, no defects consisting in uneven coloring, or the appearance of other color deviations were found. The meat raw material was therefore amenable to the curing method used, and the parameters obtained were correct.

**TABLE 3:** Influence of breed, ripening time (week) and type of garlic on color parameters of dry fermented sausages made from Polish Red and White‐Backed cattle meat with white and black garlic (ANOVA).

	Breed (B)		Ripening time (T)				Garlic (G)		Interactions			
	PR	WB	0	2	4	6	w	b	B × T	B × G	G × T	B × T × G
L*	40.09^a^	38.62^b^	44.96^a^	42.86^b^	36.16^c^	33.44^d^	39.95^a^	38.77^b^	**	*	**	**
a*	13.79^a^	13.39^b^	15.91^a^	13.87^b^	13.13^c^	11.44^d^	13.65	13.53	**	**	**	**
b*	13.13	13.23	17.54^a^	13.75^b^	11.60^c^	9.85^d^	13.85^a^	12.52^b^	**	**	**	**

*Note:* ^a,b^different letters in the rows indicate significant differences between the means at *p* < 0.05; *significant interaction at *p* < 0.05; **significant interaction at *p* < 0.01.

Abbreviations: a*, redness; b*, yellowness; L*, lightness; PR, Polish Red cattle breed; w/b, white/black garlic; WB, White‐Backed cattle breed.

**TABLE 4:** Color intensity descriptions of dry fermented sausages made from Polish Red and White‐Backed cattle meat with white and black garlic.

	PRw	PRb	WBw	WBb
C*	16.46	13.02	17.09	13.85
h°	48.13	52.68	47.36	49.85
ΔE	12.6	18.77	11.56	16.83

*Note:* sausages with Polish Red (PR)/White‐Backed (WB) cattle breed meat and white (w)/black (b) garlic; C*—chroma (the amount of saturation); h°—hue (the color tone).

The stable red color is a consequence of myoglobin nitrosylation. *Micrococcaceae* and *Staphylococcaceae* are accountable for denitrification of nitrate (V) and have a supporting function in shaping the color of cured products or are solely accountable for the colouration of raw products not cured (Baka et al. 2011; Węsierska et al. 2013). Flawed staining of sausages is feasible under conditions of advanced fat oxidation, as in the pH range of 4.5–6.0 an increase in the catalytic activity of secondary products of fat oxidation (hepta‐2,4‐dienal, 2‐nonenal, 4‐hydroxy‐2‐nonenal) and oxidation of myoglobin to green chole‐ or sulfmyoglobin is observed (Olivares et al. 2009; Wójciak et al. 2015; Węsierska, Sobolewska‐Zielińska, et al. 2021). Moreover, color deviations can also arise due to an excessive increase in the population of LAB at pH < 5.2 (Brewer and Novakofski 2008; Węsierska, Szołtysik, and Migdał 2014; Hospital et al. 2015; Degnes et al. 2017).

### 3.3 Texture of Salami‐Type Sausages Made From Meat of Two Rare Cattle Breeds

The texture of salami‐type sausages made from meat from PR and the WB shear force at the start of production was not measured due to the pasty‐like texture of the stuffing. The sausages did not differ in hardness TPA, springiness TPA, and WB shear force; however, the texture parameters changed during further stages of ripening (*p* < 0.05) (Table 5). Sausages made with black garlic were characterized by higher hardness TPA and WB shear force, but lower springiness TPA, despite comparable water, protein, and fat content (*p* < 0.05). The results were confirmed by an interaction between garlic type and ripening time (*p* < 0.01).

**TABLE 5:** Influence of breed, ripening time (week) and type of garlic on texture parameters of dry fermented sausages made from Polish Red and White‐Backed cattle meat with white and black garlic (ANOVA).

	Breed (B)		Ripening time (T)			Garlic (G)		Interactions			
	PR	WB	2	4	6	w	b	B × T	B × G	G × T	B × T × G
Hardness TPA (N)	100.55	100.71	78.14^a^	106.19^b^	117.55^c^	99.46^a^	101.80^b^			**	
Springiness TPA	0.82	0.83	0.85^a^	0.80^b^	0.82^b^	0.84^a^	0.81^b^			**	
WB sf (N/mm)	2.73	2.76	1.38^a^	2.70^b^	4.15^c^	2.45^a^	3.04^b^	*		**	

*Note:* ^a,b^different letters in the rows indicate significant differences between the means at *p* < 0.05; *significant interaction at *p* < 0.05; **significant interaction at *p* < 0.01.

Abbreviations: PR, Polish Red cattle breed; TPA, Texture Profile Analysis; w/b, white/black garlic; WB, White‐Backed cattle breed; WB sf, Warner‐Bratzler Shear Force.

The quality of dry‐cured sausages is evaluated by consumers based on texture characteristics such as hardness, chewiness, or springiness (Spaziani et al. 2009; Baka et al. 2011; Węsierska, Szmańko, and Krzysztoforski 2014; Berardo et al. 2017). The pH value, water, as well as protein, fat and salt content affect the texture of sausages. In well‐prepared products, the increase in springiness correlates with the increase in protein and salt content, and the perception of juiciness with the content of fat. Increased hardness and saltiness are characteristics of this type of product. According to Węsierska et al. (2013), the most popular fermented sausages on the market, attractive in terms of aroma, are characterized by high nutritional value (protein content 30.6%–40.6% and fat content 6.3%–12.0%), reduced water activity (0.81–0.86), and significant hardness TPA (128.6–140.3 N) and WB shear force (44.1–126.5 N).

### 3.4 Quantitative Composition of Acidifying and Denitrifying Bacteria of Salami‐Type Sausages Made From Meat of Two Rare Cattle Breeds

**FIGURE 1:**
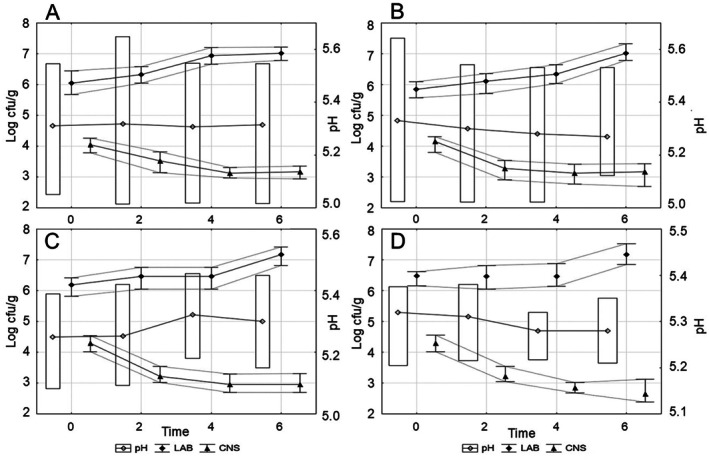
Changes in the quantitative composition of the acidifying (LAB) and denitrifying (CNS) microbiota during ripening depending on the pH of the stuffing: (A) PRw, (B) PRb, (C) WBw, (D) WBb—sausages with Polish Red (PR)/White‐Backed (WB) cattle breed meat and white (w)/black (b) garlic.

Increased salinity and reduced water activity to a value of 0.85, as well as the acidity of the stuffing at 5.3 did not inhibit the development of the acidifying microbiota, even though the development of the cocci was limited (Table 6, Figure 1). PR and WB sausages differed in the proportion of LAB (*p* < 0.05). The addition of white/black garlic also differentiated the sausage batches in terms of the amount of LAB (*p* < 0.05) and CNS (*p* < 0.05), as confirmed by interactions marked at (*p* < 0.01). In the 2nd week of ripening, the count of LAB correlated strongly and negatively with water activity values (*r* = −0.61), as did the count of staphylococci with salt content (*r* = −0.62).

**TABLE 6:** Influence of breed, ripening time (week) and type of garlic on quantitative composition of technologically desirable microbiota (log cfu/g) and environmental parameters (pH, a_w_) of dry fermented sausages made from Polish Red and White‐Backed cattle meat with white and black garlic (ANOVA).

	Breed (B)		Ripening time (T)				Garlic (G)		Interactions			
	PR	WB	0	2	4	6	w	b	B × T	B × G	G × T	B × T × G
LAB	6.53^a^	6.82^b^	6.78^a^	6.60^b^	6.12^b^	6.98^c^	6.57^a^	6.78^b^	**	**	**	**
CNS	3.27	3.28	4.12^a^	3.35^b^	2.82^c^	2.82^c^	3.32^a^	3.23^b^	**	**	**	**
pH	5.30	5.29	5.30	5.29	5.30	5.29	5.30	5.30				
a_w_	0.898	0.898	0.942^a^	0.937^b^	0.864^c^	0.850^d^	0.898	0.898	**		*	

*Note:* ^a,b^different letters in the rows indicate significant differences between the means at *p* < 0.05; *significant interaction at *p* < 0.05; **significant interaction at *p* < 0.01.

Abbreviations: a_w_, water activity; CNS, coagulase‐negative staphylococci; LAB, lactic acid bacteria; PR, Polish Red cattle breed; w/b, white/black garlic; WB, White‐Backed cattle breed.

The pH value and water activity are key factors in determining the proper orientation of biochemical transformations and the safety of fermented sausage production (Olivares et al. 2009; Marušić et al. 2013; Węsierska, Szmańko, and Krzysztoforski 2014; Degnes et al. 2017; Węsierska, Sobolewska‐Zielińska, et al. 2021). This is due to the activity of acidifying microorganisms, producing lactic acid, and endo‐ and exogenous enzyme apparatuses, releasing acidic products of biochemical transformations. Low pH characterizes cured meats ripened for a short period. According to Martin et al. (2007) and Baka et al. (2011), in sausages with longer ripening time and higher, more stable pH, the staphylococci are able to carry out at least three types of activity: (1) catabolizing free amino acids and producing flavor precursors, (2) reducing nitrate(V) and shaping the color, (3) degrading proteins to short‐chain peptides and amino acids, and then branched‐chain amino acids to aldehydes, alcohols and acids, forming the texture (Simonova et al. 2006; Spaziani et al. 2009; Latorre‐Moratalla et al. 2008; Ravyts et al. 2012). *Staphylo*‐ and *Micrococcaceae* are most eager to develop after the 2nd week of ripening, the time of the most intense fermentation of saccharides by the acidifying microbiota (Olivares et al. 2009; Węsierska et al. 2013). Fermented stuffing is a demanding environment for bacterial growth. Water loss due to drying favors groups resistant to increasing salt content (Olivares et al. 2009; Leroy et al. 2010). Technologically desirable populations therefore develop according to their ability to adapt to changing environmental conditions. At all stages of production and post‐production ripening, they are shaping the color, texture, and aroma of dry‐cured salami‐type sausages (Spaziani et al. 2009; Latorre‐Moratalla et al. 2008; Ravyts et al. 2012; Berardo et al. 2017).

### 3.5 Biogenic Amines Content of Salami‐Type Sausages Made From Meat of Two Rare Cattle Breeds

**FIGURE 2:**
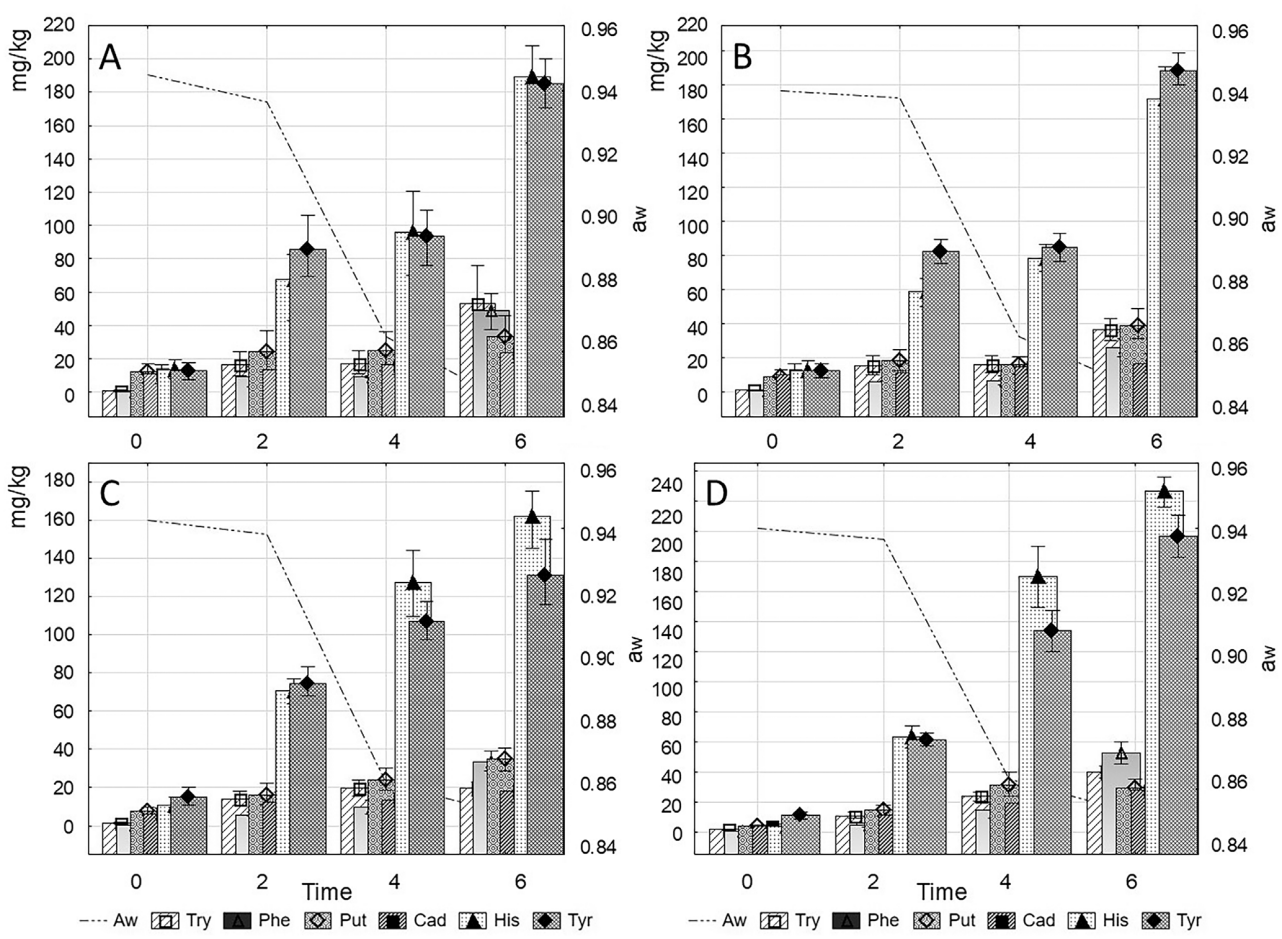
Changes in the quantitative composition of biogenic amines during ripening depending on the water activity of the stuffing: (A) PRw, (B) PRb, (C) WBw, (D) WBb—sausages with Polish Red (PR)/White‐Backed (WB) cattle breed meat and white (w)/black (b) garlic.

Significant variability was observed in the proportion of tested BAs in salami‐type sausages made from meat from PR and WB cattle breed, with the addition of white and black garlic (Table 7, Figure 2). Amine content increased during ripening (*p* < 0.05) because of the activity of the acidifying microbiota with a count of 6–7 log cfu/g. The increase in the content of the 8 analyzed BAs was influenced by the breed, the time of ripening, kind of garlic, and the interaction between all these factors (*p* < 0.01). Changes in the profile of BAs resulted from the decarboxylation of selected amino acids, for example, the higher content of histamine (*p* < 0.05) and tyramine (*p* < 0.05) in the ready‐to‐eat sausages confirmed advanced decarboxylation of histidine and tyrosine.

**TABLE 7:** Influence of breed, ripening time (week) and type of garlic on biogenic amines composition (mg/kg) of dry fermented sausages made from Polish Red and White‐Backed cattle meat with white and black garlic (ANOVA).

	Breed (B)		Ripening time (T)				Garlic (G)		Interactions			
	PR	WB	0	2	4	6	w	b	B × T	B × G	G × T	B × T × G
Try	18.48^a^	16.28^b^	1.13^a^	13.96^b^	19.09^c^	35.34^d^	16.85^a^	17.91^b^	**	**	**	**
Phe	12.71^a^	15.01^b^	0.18^a^	5.48^b^	9.80^c^	39.96^d^	14.10	13.62	**	**	**	**
Put	21.93^a^	20.15^b^	7.86^a^	18.25^b^	24.00^c^	34.05^d^	22.19^a^	19.89^b^	**	*	*	**
Cad	14.44^a^	13.91^a^	7.66^a^	11.79^b^	15.85^c^	21.37^d^	14.22	14.12	**	**	**	**
His	84.67^a^	104.27^b^	9.99^a^	65.14^b^	115.28^c^	187.46^d^	90.89^a^	98.05^b^	**	**	**	**
Tyr	97.68^a^	91.21^b^	12.71^a^	75.91^b^	104.81^c^	184.35^d^	91.38^a^	97.51^b^	**	**	**	**

*Note:* ^a,b^different letters in the rows indicate significant differences between the means at *p* < 0.05; *significant interaction at *P* < 0.05; **significant interaction at *p* < 0.01.

Abbreviations: Cad, Cadaverine; Histamine; Phe, Phenylethylamine; PR, Polish Red cattle breed; Put, Putrescine; Try, Tryptamine; Tyr, Tyramine; w/b, white/black garlic; WB, White‐Backed cattle breed.

The proportion of biogenic amines in dry‐cured meats depends on the proportion of salt and the physicochemical conditions of ripening. Increasing contribution of salt inhibits the production of some amines, such as histamine, or increases it as in the case of tyramine (Alvarez and Moreno‐Arribas 2014; Bartkiene et al. 2017). The increase in the activity of exogenous amino acid decarboxylase is observed in the pH range of 4.5–5.5, and consequently an increase in the content of amines (Lee and Kim 2013; Barbieri et al. 2019; Sivamaruthi et al. 2021; Turna et al. 2024). The addition of antioxidants inhibits the production of several biogenic amines (histamine, tyramine, putrescine) and the growth of bacteria (*Enterobacteriaceae*), responsible for their production (Latorre‐Moratalla et al. 2008; Lee and Kim 2013). Although it is a natural process inherent in the ripening of raw meat, so far, no cases of biogenic amine poisoning after consuming raw meats have been confirmed in the literature. Amine levels are also not limited by food safety criteria for raw ripened meat products (Čuboň et al. 2019). The significance of BGAs to health is ambiguous. Some can cause poisoning, although in physiological concentrations, they play important roles in vivo. However, they are also significant for food safety, and it is important that those determined in this study did not exceed levels resulting in health risks (Doeun et al. 2017; Wójcik et al. 2021). Increased tyramine content in food is a substantial problem for people who take monoamine oxidase inhibitors (about 20% of Europeans) or experience congenital impairment of this enzyme secretion. In‐taking a histamine dose of 5–10 mg with food can cause a reaction in sensitive people. Nevertheless, a dose of 10 mg is considered the acceptable limit. A dose of 100 mg is considered moderately toxic, while 1000 mg is assumed as very toxic. However, a dose 20–50 times lower may induce breathlessness attacks caused by bronchial smooth muscle spasm, swelling of the mucosa and increased secretion of mucous glands among individuals suffering from respiratory diseases. Despite the naturally occurring decarboxylation of released amino acids, no intoxication has been reported after consumption of dry‐cured sausages (Alvarez and Moreno‐Arribas 2014; Bartkiene et al. 2017; Doeun et al. 2017).

## 4 Conclusions

Organic production is associated with exorbitant consumer expectations regarding the widely understood quality of meat products. Quality can be built by selecting unique raw materials and culturally distinctive additives, health‐promoting properties, safety of the production, animal welfare and sustainable agricultural production. The use of raw materials from indigenous breeds is becoming popular, preserving cultural heritage and supporting local producers. The results indicate that both cattle breed and garlic type influence selected quality factors of fermented dry‐cured sausages. Both native cattle breeds: the Polish Red and White‐Backed are a technologically attractive to produce dry‐cured salami‐type sausages. Utility of Polish Red and White‐Backed raw materials enables to prepare a nutritionally well‐balanced meal and supplement diet with nutritionally important ingredients. The protein, fat, ash and salt content as well as the texture of ready‐to‐eat salami‐type sausages depend on the water content. As a result of drying and the increase in protein content, the hardness TPA and WB Shear Force of sausages increase. Ripening time and changing environmental conditions, including water activity and pH, affect the populations of acidifying and denitrifying microbiota, which determine, among other things, the color of ready products. Increased salinity, reduced water activity and high acidity of sausages do not inhibit the development of acidifying microbiota but limit the development of coagulase‐negative staphylococci. Decarboxylation of histidine and tyrosine indicates a high metabolic activity of microbiota under comparable conditions for all sausage types, that is, water content, water activity and pH.

## Author Contributions

**Małgorzata Pasternak:** formal analysis (equal). **Katarzyna Niemczyńska‐Wróbel:** methodology (equal).

## Conflicts of Interest

The authors declare no conflicts of interest.

## Data Availability

The data that support the findings of this study are available from the corresponding author upon reasonable request.
